# The Foreign Relations Committee

**Published:** 1899-12

**Authors:** 


### THE FOREIGN RELATIONS COMMITTEE.

THE report of the meeting of the Foreign Relations Committee, as published in the October number of the *Dental Review* and the "Circular of Information" from the same source, have been sent broadcast for the instruction of those interested. While the two reports may not find readers among the main body of dental operators, they will be read with care and attention by all those engaged in dental education, both in this country and abroad.

The report of the meeting contains not only much of interest, but the communications from the members of the several committees resident in or native of foreign countries enter very fully into the condition of dental education in their respective localities and of the laws which govern it, and if nothing else were gained, these would amply repay the labor expended in their organization. Had the Foreign Relations Committee confined its exertions to gathering information for the use of dental colleges belonging to the National Association of Dental Faculties, it would have received unlimited credit for good work.

When the Committee was appointed it was expected that dental colleges in this country would be relieved of much embarrassment in deciding questions relating to foreign applicants for advanced standing, and hence it was given all the encouragement possible at the beginning of its work, but the fear was felt by some that, possibly, we might be led into a maze of difficulties more perplexing than those which originally disturbed educational circles.

This fear has been more than realized through the two years of work of this Committee. The reason for this is not far to seek; in fact, it is clearly set forth in these two reports. The tendency of some committees is to exceed instructions, but, in the writer's experience, there have not been many that have assumed powers equal to this, never delegated to it, or one that has attempted to dictate who shall or who shall not be admitted, not only to advanced standing in American dental schools, but who may be matriculated from foreign countries in the first or Freshman year. In proof of the latter the following quotation will satisfy the critical mind. On page 2 of "Circular of Information" the Committee states that "All applications made in America for admission of foreign students to the *first year's course* of our schools must *also be referred by them to the Foreign Relations Committee for like information and approval.* This action is *imperative and will*

*henceforth be enforced upon all reputable American Dental Schools.*" (Italics ours.) The only excuse for this absurd and extravagant utterance must be found in the fact that the powers delegated to this Committee were not clearly defined by the Faculties at the time of its appointment. Reference to the rule adopted, however, will conclusively show that no such power was given this Committee, nor was it supposed by the members of the Faculties Association that it would ever be assumed. The only standing committee in that body having this power is that known as the Ad Interim Committee, elected annually by the members, and the power legitimately belonging to it will probably never be given to any other committee, especially one appointed by the president of the Association.

In the brochure before alluded to the Committee has added the rules governing the Association of Faculties. In this is the one adopted regulating the duties of the Foreign Relations Committee. It reads as follows:

"A standing committee of five shall be appointed each year by the president of this Association, to be called the Committee on Foreign Relations, whose duty it shall be to report each year upon the relative status of dentistry in America and Europe, and to suggest any measures that, in the opinion of its members, will promote the welfare of our common profession and the usefulness of the distinctive American dental degree."

The closing paragraph is as follows:

"The Foreign Relations Committee is given jurisdiction in all foreign American dental educational matters, subject always to the approval of the National Association of Dental Faculties, to which a full report shall be submitted annually."

The latter paragraph may be taken as giving authority to decide what American colleges may or may not do, but as originally intended it applies only to those making application from abroad for advanced standing, and concerns itself solely with the status of foreign applicants, and has nothing to do, directly, with American dental colleges. It is for the advising committees in the several countries to endorse or reject certificates or diplomas, and it is the duty of the proper officers of American colleges to correspond relative thereto. No self-respecting dean would think of writing to the chairman of the Foreign Relations Committee for *permission* to accept a student in the Freshman year, or even for advanced standing. What the colleges want is information, not dictatorial assumption.

In the general report of the meeting of the Foreign Relations Committee occurs the remarks of Dr. Bryan, of Basel, Switzerland. If he is correctly reported, it would seem that he should have said either less or more. The following is the paragraph as printed:

"There are fifty-two American graduates D.D.S. practising in Switzerland, and thirty-six answered a circular letter, many of whom matriculated in October and went back in June, graduated. Many do not know anything of English. Five different complaints have been filed with me of irregular practices, and all of which were against one school, and that most pretentious in its requirements."

Who gave Dr. Bryan the authority to receive complaints against American schools? Perhaps the Foreign Relations Committee can answer this query, for it certainly needs an answer.

The statement that "five complaints" were filed against one school requires explanation. Why has not that school been reported to the Ad Interim Committee? We question the correctness of so much of the statement which concerns the inability to speak English. This charge has been repeatedly made, but it has never been accompanied by attested facts. Usually the knowledge of English possessed by matriculates from Switzerland has been a constant source of surprise to the writer. It is rather singular and yet it may be true, that some school can note exceptions to this favorable opinion. By all means let us have the name of the college graduating men from Switzerland who were not conversant with the English language, spoken or written.

It is not pleasant to be forced to recount these things in a public journal, but as the Foreign Relations Committee has elected this method, there is no escape from the responsibility, and it must expect to receive the criticism its action merits.

The foreign advisory committees may yet prove a blessing to dental education in America, but if they are to accomplish this they must attend strictly to the work given them to do, and not attempt matters for which they were not appointed and for which they have no qualification. America must work out its own educational problems, and that without interference from foreign ideas which can never harmonize with those which have taken root here. We welcome opinions and advice properly given, but dental schools in Europe will have to take many steps forward before they are in a position to instruct the higher dental colleges of this country in the best methods of dental education.

When the National Association of Dental Faculties meets at Old Point Comfort, the Foreign Relations Committee will be called upon to answer to that body for an assumption of power never contemplated by that organization.

